# Impact of obesity on COVID-19 CoronaVac and ChAdOx1-S vaccine efficacy

**DOI:** 10.1186/s12879-025-12295-2

**Published:** 2025-12-09

**Authors:** Márcio Valle Cortez, Alex Martins, Joabi Nascimento, Fabíola Mendonça da Silva Chui, Maria Gabriela Almeida Rodrigues, Talita Bastos, Sonia Maria Lemos, Eduardo Honorato, Erika Gomes, Rebeca Linhares Abreu-Netto, Salete Fernandes, Alexandre Vilhena Silva-Neto, André Sachett, Bernardo Maia Silva, Gabriel Santos Mouta, Jady Shayenne Mota Cordeiro, Djane Clarys Baía-da-Silva, Jose Diego Brito-Sousa, Karina Pinheiro Pessoa, Wuelton Monteiro, Felipe Naveca, Vanderson Sampaio, Fernando Almeida-Val, Gisely Melo, Maria Paula Mourão, Marcus Lacerda

**Affiliations:** 1 Universidade do Estado do Amazonas, Manaus, Brazil; 2 Fundação de Medicina Tropical Dr Heitor Vieira Dourado, Av Pedro Teixeira 25, Manaus, Brazil; 3 Universidade Nilton Lins, Manaus, Brazil; 4 Secretaria Municipal de Saúde, Manaus, Brazil; 5 Instituto Nacional de Pesquisas da Amazônia, Manaus, Brazil; 6 Instituto Leônidas & Maria Deane, Fiocruz, Manaus Brazil; 7 Instituto Todos pela Saúde, São Paulo, Brazil; 8 University of Texas Medical Branch, Galveston, USA

**Keywords:** Obesity, COVID-19, Vaccine efficacy, CoronaVac, ChAdOx1-S, Bioimpedance, Body composition, Immunogenicity

## Abstract

**Background:**

Obesity significantly increases the risk of severe COVID-19 and mortality. Concerns have emerged regarding the efficacy of COVID-19 vaccines in obese populations due to potential alterations in immune responses.

**Methods:**

Between March 2021 and March 2022, 5,071 participants with comorbidities received CoronaVac administered in two doses 28 days (± 7 days) apart and a single subsequent booster dose of ChAdOx1 with an interval of 180 days (± 30 days). Body composition was analyzed using a tetrapolar bioimpedance measurement scale. Anti-RBD IgG was dosed at baseline (D0) and on days 28, 90, 180, 270, and 360.

**Results:**

1,181 participants with obesity (body fat percentage > P50), non-immune at baseline, showed significantly lower antibody titers (IgG anti-RBD) until D180 than 576 non-obese individuals. However, there was no difference in the frequency of COVID-19, COVID-19-related hospitalization or death. After the booster, no significant changes were seen. Patients with very excessive body fat (body fat percentage > P90) showed even slower seroconversion, with increased COVID-19 after D180. Similar results were observed using the routine body mass index (BMI); however, waning immunity (serology) was observed in individuals classified as having very excessive body fat only when bioimpedance was used.

**Conclusion:**

Participants with obesity demonstrated weaker and slower seroconversion, which worsened in higher body fat mass. Bioimpedance revealed quicker waning immunity, emphasizing its importance over BMI in assessing vaccine response.

**Supplementary Information:**

The online version contains supplementary material available at 10.1186/s12879-025-12295-2.

## Background

Obesity, defined as a body mass index (BMI) higher than 30, has emerged as a critical global health concern characterized by excessive body fat accumulation, posing significant challenges to health systems worldwide [1]. The prevalence of obesity has steadily risen over the past few decades, contributing to a range of comorbidities, including cardiovascular and metabolic diseases and respiratory disorders [2, 3]. Multiple anthropometric indicators are commonly used to evaluate obesity and its related health risks, including body mass index (BMI), waist circumference, hip circumference, waist-to-hip ratio, and waist-to-height ratio [4]. Although BMI offers a broad measure of overall adiposity, indices based on abdominal measurements more accurately capture visceral fat accumulation [5].

As the COVID-19 pandemic caused by SARS-CoV-2 evolved over the years, increasing evidence showed that obesity was a significant risk factor for severe outcomes and higher mortality rates [6–8]. The relationship between COVID-19 severity and obesity highlights the disproportionate effect on this susceptible group [9, 10], which is closely associated with impaired lung function, dysregulated immune responses, and chronic inflammation, all of which increase the risk of respiratory infections and worsen clinical outcomes [11].

Epidemiological studies have consistently demonstrated a dose-response relationship between increasing BMI and the risk of severe COVID-19 outcomes, including the need for hospitalization, mechanical ventilation, and mortality [12, 13]. Moreover, obese individuals often present with underlying conditions such as hypertension, type 2 diabetes, and compromised immune function, further compounding the risks associated with COVID-19 infection [3, 14].

Vaccination emerged as a pivotal tool in controlling the spread of COVID-19 and mitigating its impact on public health [2, 15]. While increased immune boosting is noted in normal-weight patients [16], concerns have been raised regarding the efficacy of COVID-19 vaccines in obese populations, given the potential for altered immune responses and vaccine effectiveness. Although evidence suggests that humoral responses to COVID-19 vaccines may be reduced in individuals with overweight or obesity, conclusive data on vaccine safety in this population are lacking [17]. Understanding the nuances of seroconversion in the context of obesity is paramount for optimizing vaccine strategies and ensuring equitable access to effective COVID-19 immunization programs. Hence, this study evaluated the immune response of participants with obesity who received two doses of the CoronaVac vaccine with a booster dose of the AstraZeneca vaccine in Manaus, Brazil.

## Methods

### Study design

This observational study was part of the CovacManaus I and II, assessed the effectiveness of the adsorbed inactivated COVID-19 vaccine CoronaVac (D0 and D28) with a booster dose of the AstraZeneca vaccine (D180) in Manaus, Brazil (NCT04789356/NCT05289206). In the study context, participants with a higher risk of infection and severity (defined by the national plan but not yet the priority group) anticipated the vaccine, which was only available to older groups during the rollout campaign in Brazil due to limited supply. No randomization was performed, and participants were allocated to intervention using a risk-based allocation. Details of the CovacManaus trial will be published elsewhere.

### Population

Participants were screened virtually, with a scheduled in-person appointment to confirm eligibility criteria at two sites in Manaus between March 2021 and March 2022. For this analysis, inclusion criteria were (a) age between 18 and 49 years; (b) presence of at least one comorbidity listed in the Brazilian National Immunization Plan; and (c) willingness to be monitored during the follow-up period defined in the study through visits, telephone contacts, or other means of digital communication. Exclusion criteria were (a) confirmed diagnosis of COVID-19 in the previous 28 days (antigen test or RT-PCR), in this case, vaccination could be delayed until 30 days later; (b) report of fever in the 72 h before vaccination (inclusion could be delayed until the participant had been fever-free for 72 h and COVID-19 has been ruled out); (c) have received a live attenuated vaccine in the last 28 days or an inactivated vaccine in the previous 14 days before inclusion in the study; (d) Participants were excluded if they had any clinically significant condition that, in the opinion of the investigator, could increase the risk associated with study participation or interfere with the interpretation of safety data—such as unstable chronic illnesses, significant immunosuppression, poorly controlled metabolic or autoimmune diseases, a history of serious vaccine-related adverse reactions, or any medical, psychiatric, or social factor likely to compromise safety or follow-up; (e) pregnancy or lactation and (f) failure to meet any inclusion criteria.

### Procedures and interventions

After a review of eligibility, demographic information, pre-existing medical conditions, and contact information were collected. Blood samples were collected before each vaccine dose and during follow-up visits (D0, D28, D90, D180, D270, and D360).

BMI was calculated on D0, and tetrapolar bioimpedance analysis (BIA) was performed on D28 (for logistical reasons, but it was used as a proxy for the exam performed before inclusion). BIA was carried out using an InBody 270 (Biospace, California, USA), and body fat (BF) levels were classified following a normative table for BF percentages validated in Brazilian adults per gender [18] (Supplementary Table 1). Subjects were classified as **above normal body fat (BF)/obese (>P50)** and **normal BF/non-obese (< P50)**, and as **very excessive BF (>P90)** and **non-very excessive BF (< P90)**, through BIA, and following percentiles used for the Brazilian population.

Nasopharyngeal swabs were collected for COVID-19 confirmation through RT-PCR during scheduled visits and through passive surveillance whenever the participant reported any symptoms to the study team.

The CoronaVac (Sinovac Biotech, China) vaccine was administered in two doses, 28 days (± 7 days) apart, via intramuscular injection using 25 mm needles. A single booster dose of ChAdOx1 vaccine (Oxford-AztraZeneca) was administered intramuscularly in the deltoid, using 30 mm needles, with an interval of 180 days (± 30 days) apart from the second dose of the CoronaVac. Total antibodies against the viral nucleocapsid and IgG anti-RBD (anti-receptor-binding domain) antibodies against the Spike protein were measured using the commercial kits (Elecsys Anti-SARS-CoV-2 Total and Elecsys Anti-SARS-CoV-2). The results are expressed in U/mL, and samples are categorized as reactive (> 0.8 U/mL) or highly reactive (≥ 250 U/mL) for SARS-CoV-2 RBD-specific antibodies.

### Outcomes

Outcomes were the titration of antibodies against SARS-CoV-2 (IgG Anti-RBD) on D28, D90, D180, D270, and D360 post-vaccination in participants without previous immunity (cut point < 0.8 U/mL at D0). Other outcomes included the development of COVID-19, the need for hospitalization attributed to COVID-19, and death attributed to COVID-19 at any follow-up time point.

### Statistical analysis

The proportions between the groups were compared using the chi-square test and Fisher’s exact test for categorical variables. Mean and standard deviation, median, and interquartile ranges were calculated for continuous variables and analyzed using ANOVA and Wilcoxon (two groups). All analyses were carried out using Stata (v.17).

## Results

Between March 2021 and March 2022, 5,071 participants were included, and 1,757 had non-reactive IgG levels at baseline. Most were females (57.4%), with a median age of 40 years (IQR 35.0–45.0), and of admixed race (62.9%). Table 1 summarizes demographic data. Overall, the non-obese group had a higher proportion of reactive IgG at D28 and highly reactive IgG on D180 than the participants with obesity. No difference in disease, hospitalization, and death rates was found at any follow-up timepoint (Table 2).

**Table 1:** Baseline demographic data

	Total	Normal BF	Above normal BF
	*N* = 1757	*n* = 576	*n* = 1181
Gender Women, %	1009/1757 (57.43)	423/576 (73.44)	586/1181 (49.62)
Age, Median (IQR)	40.0 (35.0–45.0)	42.0 (37.0–46.0)	39.0 (34.0–44.0)
Race, %			
White	460/1757 (26.18)	131/576 (22.74)	329/1181 (27.86)
Black	144/1757 (8.20)	47/576 (8.16)	97/1181 (8.21)
Admixed	1105/1757 (62.89)	378/576 (65.63)	727/1181 (61.56)
Asian	31/1757 (1.76)	13/576 (2.26)	18/1181 (1.52)
Indigenous	12/1757 (0.68)	5/576 (0.87)	7/1181 (0.59)
BMI, Median (IQR)	32.30 (29.60–36.10)	28.00 (25.00-30.10)	34.30 (32.10–38.20)
Weight in Kg, Median (IQR)	88.1 (76.9-100.2)	71.9 (64.6–78.0)	95.2 (87.0-106.6)
Height in in centimeters, Median (IQR)	164.0 (158.0-172.0)	160.0 (155.0-167.0)	166.0 (159.0-173.0)
Diabetes, %	170/1757 (9.68)	70/576 (12.15)	100/1181 (8.47)
Severe chronic lung disease, %	110/1757 (6.26)	73/576 (12.67)	37/1181 (3.13)
Systemic arterial hypertension, %	379/1757 (21.57)	187/576 (32.47)	192/1181 (16.26)
Cardiovascular diseases, %	32/1757 (1.82)	25/576 (4.34)	7/1181 (0.59)
Cerebrovascular disease, %	3/1757 (0.17)	2/576 (0.35)	1/1181 (0.08)
Chronic kidney disease, %	6/1757 (0.34)	5/576 (0.87)	1/1181 (0.08)
Immunosuppressed, %	98/1757 (5.58)	79/576 (13.72)	19/1181 (1.61)
Sickle cell anemia, %	1/1757 (0.06)	1/576 (0.17)	0/1181 (0.00)

*BF*, body fat.* D*, day.* IQR*, interquartile range

**Table 2:** Seroconversion (IgG anti-RBD) in participants without previous immunity between D0 and D360 in participants with normal and above normal body fat

	Total	Normal BF	Above normal BF	*p*
	*N =* 1757	*n =* 576	*n =* 1181	
**D0 to D28**				
COVID-19, %	42/1757 (2.39)	11/576 (1.91)	31/1181 (2.62)	0.36
Hospitalization by COVID-19, %	1/1757 (0.06)	0/576 (0.00)	1/1181 (0.08)	1.00
Reactive serology in D28 (> 0.8 U/mL), %	1345/1741 (77.25)	464/571 (81.26)	881/1170 (75.30)	**0.005**
Reactive serology in D28 (≥ 250 U/mL), %	9/1741 (0.52)	3/571 (0.53)	6/1170 (0.51)	0.97
**D0 to D90**				
COVID-19, %	117/1757 (6.66)	44/576 (7.64)	73/1181 (6.18)	0.25
Hospitalization by COVID-19, %	2/1757 (0.11)	0/576 (0.00)	2/1181 (0.17)	1.00
Reactive serology in D90 (> 0.8 U/mL), %	1626/1637 (99.33)	533/537 (99.26)	1093/1100 (99.36)	0.80
Reactive serology in D90 (≥ 250 U/mL), %	230/1637 (14.05)	85/537 (15.83)	145/1100 (13.18)	0.15
**D0 to D180**				
COVID-19, %	122/1757 (6.94)	41/576 (7.12)	81/1181 (6.86)	0.84
Hospitalization by COVID-19, %	5/1757 (0.28)	0/576 (0.00)	5/1181 (0.42)	0.18
Reactive serology in D180 (> 0.8 U/mL), %	1603/1615 (99.26)	537/541 (99.26)	1066/1074 (99.26)	0.99
Reactive serology in D180 (≥ 250 U/mL), %	211/1615 (13.07)	88/541 (16.27)	123/1074 (11.45)	**0.007**
**D0 to D270**				
COVID-19, %	713/1757 (40.58)	238/576 (41.32)	475/1181 (40.22)	0.66
Hospitalization by COVID-19, %	6/1757 (0.34)	0/576 (0.00)	6/1181 (0.51)	0.19
Reactive serology in D180 (> 0.8 U/mL), %	1358/1359 (99.93)	466/467 (99.79)	892/892 (100.00)	0.17
Reactive serology in D180 (≥ 250 U/mL), %	1319/1359 (97.06)	450/467 (96.36)	869/892 (97.42)	0.27
**D0 to D360**				
COVID-19, %	736/1757 (41.89)	248/576 (43.06)	488/1181 (41.32)	0.49
Hospitalization by COVID-19, %	6/1757 (0.34)	0/576 (0.00)	6/1181 (0.51)	0.19
Reactive serology in D360 (> 0.8 U/mL), %	1016/1016 (100.00)	368/368 (100.00)	648/648 (100.00)	--
Reactive serology in D360 (≥ 250 U/mL), %	1007/1016 (99.11)	364/368 (98.91)	643/648 (99.23)	0.61

*BF*, body fat*. D*, day

When further subclassifying participants, less reactive antibodies were seen in very excessive BF as compared to non-very excessive BF on D28, and less highly reactive were seen in D90 and D360. When following participants until D270 or D360, very excessive BF presented with more clinical COVID-19, however, without any impact on hospitalization and death (Table 3). When BMI was used, similar results were seen, except when subgrouping participants with severe obesity (BMI < 40 vs. ≥ 40), in which no difference in disease and seroconversion was observed after D180 (supplementary Tables 2 and 3). No differences were observed when grouping patients per visceral fat level (supplementary Table 4).

**Table 3:** Seroconversion (IgG anti-RBD) in participants without previous immunity between D0 and D360 in very excessive vs. non-very excessive BF

	Total	Non-very excessive BF	Very excessive BF	*p*
	*N =* 1757	*n =* 1409	*n =* 348	
**D0 to D28**				
COVID-19, %	42/1757 (2.39)	33/1409 (2.34)	9/348 (2.59)	0.79
Hospitalization by COVID-19, %	1/1757 (0.06)	1/1409 (0.07)	0/348 (0.00)	1.00
Reactive serology in D28 (> 0.8 U/mL), %	1345/1741 (77.25)	1113/1398 (79.61)	232/343 (67.64)	**< 0.001**
Reactive serology in D28 (≥ 250 U/mL), %	9/1741 (0.52)	8/1398 (0.57)	1/343 (0.29)	0.52
**D0 to D90**				
COVID-19, %	117/1757 (6.66)	91/1409 (6.46)	26/348 (7.47)	0.50
Hospitalization by COVID-19, %	2/1757 (0.11)	2/1409 (0.14)	0/348 (0.00)	1.00
Reactive serology in D90 (> 0.8 U/mL), %	1626/1637 (99.33)	1309/1316 (99.47)	317/321 (98.75)	0.16
Reactive serology in D90 (≥ 250 U/mL), %	230/1637 (14.05)	200/1316 (15.20)	30/321 (9.35)	**0.007**
**D0 to D180**				
COVID-19, %	122/1757 (6.94)	94/1409 (6.67)	28/348 (8.05)	0.37
Hospitalization by COVID-19, %	5/1757 (0.28)	3/1409 (0.21)	2/348 (0.57)	0.26
Reactive serology in D180 (> 0.8 U/mL), %	1603/1615 (99.26)	1285/1293 (99.38)	318/322 (98.76)	0.24
Reactive serology in D180 (≥ 250 U/mL), %	211/1615 (13.07)	175/1293 (13.53)	36/322 (11.18)	0.26
**D0 to D270**				
COVID-19, %	713/1757 (40.58)	554/1409 (39.32)	159/348 (45.69)	**0.030**
Hospitalization by COVID-19, %	6/1757 (0.34)	3/1409 (0.21)	3/348 (0.86)	0.096
Reactive serology in D180 (> 0.8 U/mL), %	1358/1359 (99.93)	1098/1099 (99.91)	260/260 (100.00)	0.63
Reactive serology in D180 (≥ 250 U/mL), %	1319/1359 (97.06)	1066/1099 (97.00)	253/260 (97.31)	0.79
**D0 to D360**				
COVID-19, %	736/1757 (41.89)	574/1409 (40.74)	162/348 (46.55)	**0.049**
Hospitalization by COVID-19, %	6/1757 (0.34)	3/1409 (0.21)	3/348 (0.86)	0.096
Reactive serology in D360 (> 0.8 U/mL), %	1016/1016 (100.00)	831/831 (100.00)	185/185 (100.00)	--
Reactive serology in D360 (≥ 250 U/mL), %	1007/1016 (99.11)	826/831 (99.40)	181/185 (97.84)	**0.041**

*BF*, body fat*. D*, day

## Discussion

In this study, participants with obesity exhibited slower and weaker early immune responses than their comparators. Additionally, those with severe obesity experienced differences in the durability of their long-term immune response, with an accelerated waning of humoral response and clinical disease, which was only evidenced using BIA.

COVID-19 patients typically produce antibodies within the first weeks after symptom onset and remain elevated for several months [19, 20]. Although COVID-19 vaccination is highly effective in eliciting a protective humoral response, antibody titers may be lower in individuals with obesity compared to the general population [21, 22], and the decline in antibody titers over time may also be associated with increased morbidity and mortality [21]. The influence of other conditions, such as chronic kidney disease, diabetes, and hypertension, usually associated with obesity, may also explain the heterogeneity of COVID-19 vaccine response [23–26]. Reduced immunogenicity in overweight patients has also been observed in vaccines for other diseases [27, 28].

Vaccination may also play a protective role against the development and severity of long COVID, primarily through the reduction of viral burden and modulation of the inflammatory signaling pathways implicated in post-acute sequelae [29, 30]. Also, while conventional vaccine platforms have demonstrated substantial success in COVID-19, novel technologies such as circular RNA vaccines may be alternatives with potential advantages in stability and immunogenicity [31].

The length of the needle used for vaccination may affect vaccine delivery in individuals with obesity [32, 33]. The thicker subcutaneous fat layer hinders proper delivery into muscle tissue, potentially compromising vaccine absorption and efficacy. After a preliminary analysis, and with the possibility that smaller needles could have impacted the adequate immunization of the obese population because of the fat layer in front of the muscle, a larger needle was used for the booster dose administration.

The group with very high body fat levels exhibited waning immunity over time, even after receiving a booster dose—an effect not observed with similar BMI classifications. While BMI has been widely used to categorize nutritional status, it only approximates the degree of adiposity and fails to account for body fat distribution, particularly visceral fat. Consequently, individuals with high muscle mass may be inaccurately classified as overweight, whereas those with low lean mass and increased adiposity may be misclassified as average weight. In contrast, body composition analysis through bioimpedance measurement offers a more accurate assessment of lean body mass and body fat in clinical evaluations [34]. Research indicates visceral adiposity is a more sensitive predictor of adverse COVID-19 outcomes than BMI alone [35]. While negative correlations between BMI and serum levels of SARS-CoV-2 Spike-specific IgG antibodies have also been documented [36, 37], and bioimpedance may not always be readily available in clinical settings, it highlights the importance of employing more precise metrics in understanding the immune response in obese individuals that would otherwise not be seen using BMI.

This study had limitations. Due to its design, it may be unclear whether the vaccine or possible infections during follow-up elicited immunity in participants with obesity. Although comorbidities were recorded, we could not assess their effects from the outcomes assessed. Residual confounding related to underlying conditions is therefore possible, particularly because some comorbidities may influence immune response, clinical evolution, or treatment effectiveness. An additional limitation is the potential influence of SARS-CoV-2 lineage evolution over the study period [38]. The emergence and circulation of distinct viral variants may have affected infection rates, hospitalization, and mortality independently of vaccine or host-related factors. The cellular response was not assessed in this study. Further dilutions in antibody titrations could not be performed when levels were ≥ 250 U/mL.

Additionally, this study also has several strengths. It included a large cohort with prospective follow-up and standardized vaccination schedules using two vaccine platforms. The use of serial serological measurements across multiple time points allowed for the assessment of antibody kinetics over time. Additionally, body composition was evaluated through tetrapolar bioimpedance, providing a more accurate estimate of adiposity than traditional BMI alone.

In conclusion, our study demonstrated that participants with obesity with no previous immunity exhibited slower and weaker seroconversion rates following COVID-19 vaccination. Importantly, we found that waning immunity was accelerated in individuals classified as having severe obesity based on bioimpedance measures, a pattern not observed with traditional BMI classifications. Given that both short-term and long-term seroconversion may be compromised in this population, there is a critical need for targeted vaccine prioritization policies to ensure adequate protection for obese individuals.

## Supplementary information

Below is the link to the electronic supplementary material.

Supplementary Material 1

## Data Availability

The datasets used and analyzed during the current study are available from the corresponding author upon reasonable request.

## Abbreviations

ANOVA: Analysis of variance
BF: Body fat
BIA: Bioimpedance analysis
BMI: Body mass index
COVID-19: Coronavirus disease 2019
IgG: Immunoglobulin G
IQR: Interquartile range
RBD: Receptor-binding domain of SARS-CoV-2 spike protein
RT-PCR: Reverse transcription polymerase chain reaction
SARS-CoV-2: Severe acute respiratory syndrome coronavirus 2
U/mL: Units per milliliter
