# Supplementary material for: Impact of obesity on COVID-19 CoronaVac and ChAdOx1-S vaccine efficacy
Source: BMC Infect Dis. 2025 Dec 9;26:57. doi: 10.1186/s12879-025-12295-2 (PMC12801550; doi:10.1186/s12879-025-12295-2)

Supplementary tables

Supplementary Table 1. Cutoff points of body fat percentages in Brazilian adults [18].

	<P50 Normal	>P50 Above normal	>P75 Excessive	>P90 Very Excessive	>P97 Extremely excessive
Men (body fat %)	17.6–25.3	25.4–35.1	35.2–43.0	43.1–49.4	≥49.5
Women (body fat %)	28.8–35.7	35.8–42.9	43.0–49.1	49.2–52.1	≥52.2

Supplementary Table 2. Seroconversion by participant group classified as obese (BMI>30) and non-obese (<30) without previous immunity.

	Total *N*=1757	BMI<30 *n*=506	BMI>30 *n*=1251	*p*
**D0 to D28**				
COVID-19, %	42/1757 (2.39)	7/506 (1.38)	35/1251 (2.80)	0.079
Hospitalization by COVID-19, %	1/1757 (0.06)	0/506 (0.00)	1/1251 (0.08)	1.00
Reactive serology in D28 (>0.8 U/mL), %	1345/1741 (77.25)	407/501 (81.24)	938/1240 (75.65)	**0.012**
Reactive serology in D28 (≥250 U/mL), %	9/1741 (0.52)	3/501 (0.60)	6/1240 (0.48)	0.76
**D0 to D90**				
COVID-19, %	117/1757 (6.66)	33/506 (6.52)	84/1251 (6.71)	0.88
Hospitalization by COVID-19, %	2/1757 (0.11)	0/506 (0.00)	2/1251 (0.16)	1.00
Reactive serology in D90 (>0.8 U/mL), %	1626/1637 (99.33)	469/472 (99.36)	1157/1165 (99.31)	0.91
Reactive serology in D90 (≥250 U/mL), %	230/1637 (14.05)	79/472 (16.74)	151/1165 (12.96)	**0.046**
**D0 to D180**				
COVID-19, %	135/1757 (7.68)	35/506 (6.92)	100/1251 (7.99)	0.44
Hospitalization by COVID-19, %	5/1757 (0.28)	0/506 (0.00)	5/1251 (0.40)	0.33
Reactive serology in D180 (>0.8 U/mL), %	1603/1615 (99.26)	474/477 (99.37)	1129/1138 (99.21)	0.73
Reactive serology in D180 (≥250 U/mL), %	211/1615 (13.07)	75/477 (15.72)	136/1138 (11.95)	**0.040**
**D0 to D270**				
COVID-19, %	714/1757 (40.64)	203/506 (40.12)	511/1251 (40.85)	0.78
Hospitalization by COVID-19, %	6/1757 (0.34)	0/506 (0.00)	6/1251 (0.48)	0.19
Reactive serology in D270 (>0.8 U/mL), %	1358/1359 (99.93)	416/417 (99.76)	942/942 (100.00)	0.13
Reactive serology in D270 (≥250 U/mL), %	1319/1359 (97.06)	402/417 (96.40)	917/942 (97.35)	0.34
**D0 to D360**				
COVID-19, %	736/1757 (41.89)	211/506 (41.70)	525/1251 (41.97)	0.92
Hospitalization by COVID-19, %	6/1757 (0.34)	0/506 (0.00)	6/1251 (0.48)	0.19
Reactive serology in D360 (>0.8 U/mL), %	1016/1016 (100.00)	321/321 (100.00)	695/695 (100.00)	--
Reactive serology in D360 (≥250 U/mL), %	1007/1016 (99.11)	319/321 (99.38)	688/695 (98.99)	0.54

BMI, body mass index.

Supplementary Table 3. Seroconversion by groups classified as morbid (BMI≥40) and non-morbid (<40) obesity, without previous immunity.

**D0 to D28**	Total *N*=1757	BMI<40 *n*=1558	BMI≥40 *n*=199	*p*
Covid-19, %	42/1757 (2.39)	39/1558 (2.50)	3/199 (1.51)	0.39
Hospitalization by COVID-19, %	1/1757 (0.06)	1/1558 (0.06)	0/199 (0.00)	1.00
Reactive serology in D28 (>0.8 U/mL), %	1345/1741 (77.25)	1215/1544 (78.69)	130/197 (65.99)	**<0.001**
Reactive serology in D28 (≥250 U/mL), %	9/1741 (0.52)	8/1544 (0.52)	1/197 (0.51)	0.98
**D0 to D90**				
COVID-19, %	117/1757 (6.66)	105/1558 (6.74)	12/199 (6.03)	0.71
Hospitalization by COVID-19, %	2/1757 (0.11)	2/1558 (0.13)	0/199 (0.00)	1.00
Reactive serology in D90 (>0.8 U/mL), %	1626/1637 (99.33)	1452/1459 (99.52)	174/178 (97.75)	**0.006**
Reactive serology in D90 (≥250 U/mL), %	230/1637 (14.05)	215/1459 (14.74)	15/178 (8.43)	**0.022**
**D0 to D180**				
COVID-19, %	135/1757 (7.68)	122/1558 (7.83)	13/199 (6.53)	0.52
Hospitalization by COVID-19, %	5/1757 (0.28)	5/1558 (0.32)	0/199 (0.00)	1.00
Reactive serology in D180 (>0.8 U/mL), %	1603/1615 (99.26)	1422/1430 (99.44)	181/185 (97.84)	**0.017**
Reactive serology in D180 (≥250 U/mL), %	211/1615 (13.07)	193/1430 (13.50)	18/185 (9.73)	0.15
**D0 to D270**				
COVID-19, %	714/1757 (40.64)	627/1558 (40.24)	87/199 (43.72)	0.35
Hospitalization by COVID-19, %	6/1757 (0.34)	5/1558 (0.32)	1/199 (0.50)	0.51
Reactive serology in D270 (>0.8 U/mL), %	1358/1359 (99.93)	1215/1216 (99.92)	143/143 (100.00)	0.73
Reactive serology in D270 (≥250 U/mL), %	1319/1359 (97.06)	1181/1216 (97.12)	138/143 (96.50)	0.68
**D0 to D360**				
COVID-19, %	736/1757 (41.89)	647/1558 (41.53)	89/199 (44.72)	0.39
Hospitalization by COVID-19, %	6/1757 (0.34)	5/1558 (0.32)	1/199 (0.50)	0.51
Reactive serology in D360 (>0.8 U/mL), %	1016/1016 (100.00)	915/915 (100.00)	101/101 (100.00)	--
Reactive serology in D360 (≥250 U/mL), %	1007/1016 (99.11)	908/915 (99.23)	99/101 (98.02)	0.22

BMI, body mass index.

Supplementary table 4. Seroconversion by groups classified per visceral fat levels, without previous immunity.

	Visceral Fat Level			
	Total<br>*N*=1757	Healthy<br>*n*=176	High (increased risk)<br>*n*=1581	*P*
**D0 to D28**				
COVID-19, %	42/1757 (2.39)	3/176 (1.70)	39/1581 (2.47)	0.79
Hospitalization by COVID-19, %	1/1752 (0.06)	0/176 (0.00)	1/1576 (0.06)	1.00
Reactive serology in D28 (>0.8 U/mL), %	1345/1741 (77.25)	133/176 (75.57)	1212/1565 (77.44)	0.57
Reactive serology in D28 (≥250 U/mL), %	9/1741 (0.52)	0/176 (0.00)	9/1565 (0.58)	0.61
**D0 to D90**				
COVID-19, %	117/1757 (6.66)	16/176 (9.09)	101/1581 (6.39)	0.17
Hospitalization by COVID-19, %	2/1731 (0.12)	0/173 (0.00)	2/1558 (0.13)	1.00
Reactive serology in D90 (>0.8 U/mL), %	1626/1637 (99.33)	155/156 (99.36)	1471/1481 (99.32)	0.96
Reactive serology in D90 (≥250 U/mL), %	230/1637 (14.05)	19/156 (12.18)	211/1481 (14.25)	0.48
**D0 to D180**				
COVID-19, %	122/1756 (6.95)	15/175 (8.57)	107/1581 (6.77)	0.37
Hospitalization by COVID-19, %	5/1712 (0.29)	0/172 (0.00)	5/1540 (0.32)	1.00
Reactive serology in D180 (>0.8 U/mL), %	1603/1615 (99.26)	167/168 (99.40)	1436/1447 (99.24)	0.81
Reactive serology in D180 (≥250 U/mL), %	211/1615 (13.07)	23/168 (13.69)	188/1447 (12.99)	0.80
**D0 to D270**				
COVID-19, %	713/1757 (40.58)	73/176 (41.48)	640/1581 (40.48)	0.80
Hospitalization by COVID-19, %	6/1757 (0.34)	0/176 (0.00)	6/1581 (0.38)	1.00
Reactive serology in D270 (>0.8 U/mL), %	1358/1359 (99.93)	142/142 (100.00)	1216/1217 (99.92)	0.73
Reactive serology in D270 (≥250 U/mL), %	1319/1359 (97.06)	136/142 (95.77)	1183/1217 (97.21)	0.34
**D0 to D360**				
COVID-19, %	736/1757 (41.89)	77/176 (43.75)	659/1581 (41.68)	0.60
Hospitalization by COVID-19, %	6/1757 (0.34)	0/176 (0.00)	6/1581 (0.38)	1.00
Reactive serology in D360 (>0.8 U/mL), %	1016/1016 (100.00)	106/106 (100.00)	910/910 (100.00)	--
Reactive serology in D360 (≥250 U/mL), %	1007/1016 (99.11)	106/106 (100.00)	901/910 (99.01)	0.30

Visceral Fat Level : healthy (<10) and high (≥10)

Supplementary Figure 1. Antibody titers during follow-up in participants without previous immunity.

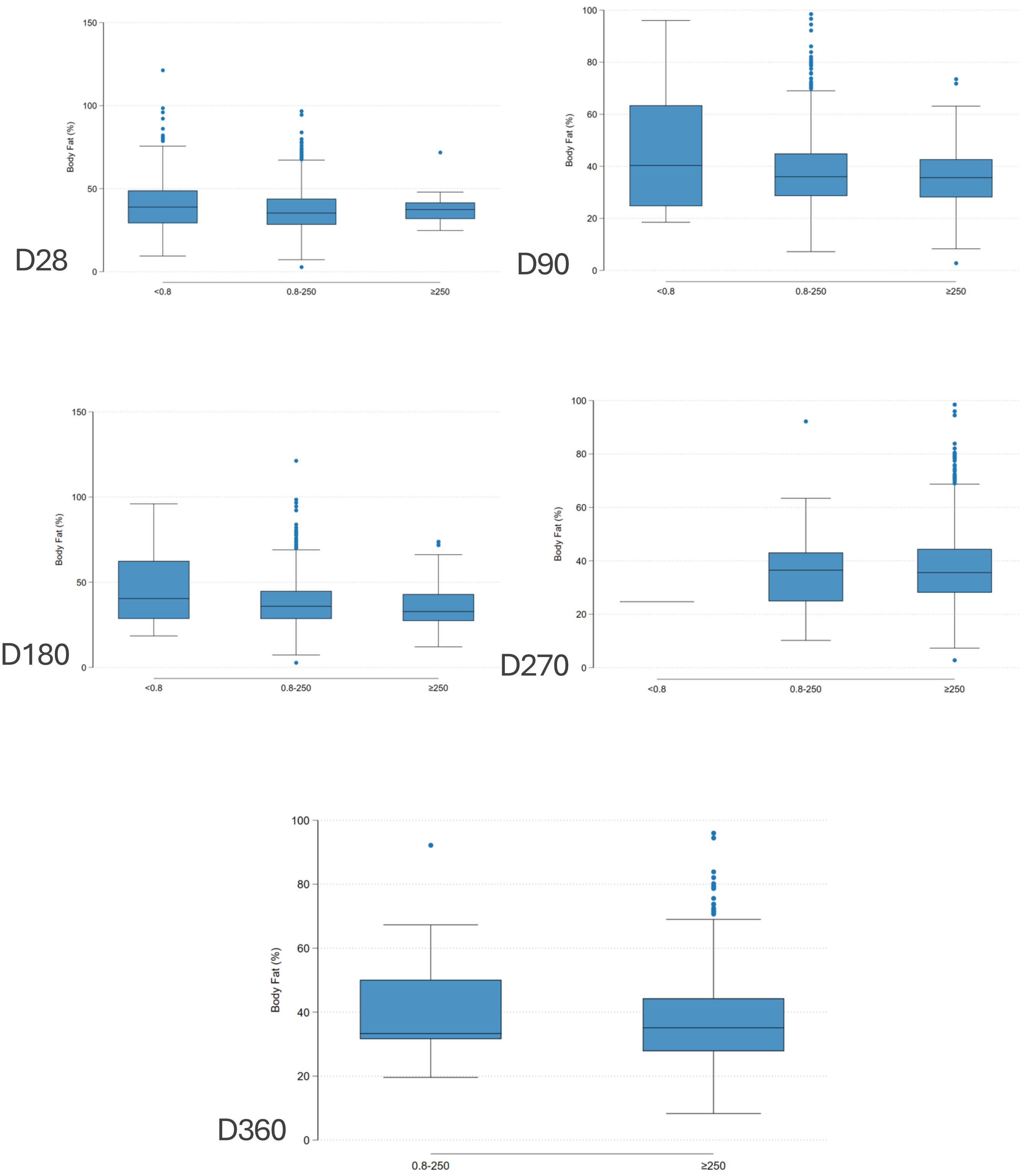

Supplement: Supplementary file 1 — Supplementary Material 1 [file 12879_2025_12295_MOESM1_ESM.pdf]
